# Fatty acid distribution of cord and maternal blood in human pregnancy: special focus on individual *trans *fatty acids and conjugated linoleic acids

**DOI:** 10.1186/1476-511X-10-247

**Published:** 2011-12-30

**Authors:** Uta Enke, Anke Jaudszus, Ekkehard Schleussner, Lydia Seyfarth, Gerhard Jahreis, Katrin Kuhnt

**Affiliations:** 1 Placenta Laboratory, Department of Obstetrics, Jena University Hospital, Friedrich Schiller University Jena, Bachstrasse 18, Jena, Germany; 2 Department of Nutritional Physiology, Institute of Nutrition, Friedrich Schiller University Jena, Dornburger Str. 24, Jena, Germany

**Keywords:** *trans *octadecenoic acids, *trans *fatty acids, vaccenic acid, elaidic acid, conjugated linoleic acids, omega-3 LC-PUFA, *t*9/*t*11-index, fetus, pregnancy, dairy fat

## Abstract

**Background:**

Maternal nutrition in pregnancy has a crucial impact on the development of the fetus. Dietary *trans *fatty acids (*t*FA) are known to have adverse health effects, especially during pregnancy. However, the distribution of *t*FA produced via partial hydrogenation of vegetable oils (mainly elaidic acid; *t*9) differs compared to ruminant-derived *t*FA (mainly vaccenic acid; *t*11). Recent findings indicate that they may have different impact on human health.

Therefore, in this study, plasma and erythrocytes of mother-child pairs (n = 55) were sampled to investigate the distribution of *t*FA, including individual *trans *C18:1 fatty acids and conjugated linoleic acids (CLA) in fetal related to maternal lipids; with additional consideration of maternal dairy fat intake.

**Results:**

Portion of *t*9 and *t*11, but also of *c*9,*t*11 CLA was higher in maternal than in fetal blood lipids. The portion of *t*9 in maternal and fetal lipids differed only slightly. In contrast, the portion of fetal *t*11 was only half of that in maternal blood. This led to a fetal *t*9/*t*11-index in plasma and erythrocytes being twice as high compared to the maternal values. A high dairy fat intake resulted in elevated portions of *t*11 and its Δ9-desaturation product *c*9,*t*11 CLA in maternal blood. In contrast, in the respective fetal blood lipids only *c*9,*t*11 CLA, but not *t*11 was increased. Nevertheless, a positive association between maternal and fetal plasma exists for both *t*11 and *c*9,*t*11 CLA. Furthermore, in contrast to *t*9, *t*11 was not negatively associated with n-3 LC-PUFA in fetal blood lipids.

**Conclusions:**

Fetal blood fatty acid composition essentially depends on and is altered by the maternal fatty acid supply. However, in addition to dietary factors, other aspects also contribute to the individual fatty acid distribution (oxidation, conversion, incorporation). The lower portion of fetal *t*11 compared to maternal *t*11, possibly results from Δ9-desaturation to *c*9,*t*11 CLA and/or oxidation. Based on the fatty acid distribution, it can be concluded that *t*11 differs from *t*9 regarding its metabolism and their impact on fetal LC-PUFA.

## Background

Maternal nutrition may have a crucial impact on the development of the fetus during pregnancy and even for the child after birth [1]. Long chain polyunsaturated fatty acids (LC-PUFA), such as C20:4 n-6 (arachidonic acid; AA) and C22:6 n-3 (docosahexaenoic acid; DHA) are known to be essential for fetal development [2-4]. Moreover, n-3 LC-PUFA have been shown to modulate the fetal immune system and, thus, possibly decrease the risk for later atopy development [5]. In contrast, *trans *fatty acids (*t*FA) are considered to enhance the risk of coronary heart disease [6,7], atopy [8], complications in pregnancy, e.g. preeclampsia [9] and to impair fetal development [10]. Extensive data on preferred LC-PUFA transport from mother to fetus [11,12] as well as higher LC-PUFA concentrations in fetal than in maternal blood [13-16] have been published over the last few decades. In contrast, data of fetal blood concentrations and profile of conjugated linoleic acids (CLA) and *t*FA are scarce. Moreover, in results from studies concerning *t*FA, only mean percentage values of total *t*FA in maternal and/or fetal plasma and erythrocyte lipids have been reported [13,17-20].

However, since the amount and distribution of individual *t*FA, especially *trans *C18:1, differs considerably between industrial and natural (ruminant) *t*FA sources, a discussion has arisen regarding the extent to which ruminant *t*FA raises the risk for cardiovascular diseases in comparison to industrial *t*FA. The latter mainly comprises of *trans-*9 C18:1 (elaidic acid; *t*9) and *trans-*10 C18:1 [21]. In contrast, *trans-*11 C18:1 (vaccenic acid; *t*11) is the major *trans *C18:1 in ruminant *t*FA principally resulting in a low *t*9/*t*11-index in dairy products (< 1) [22]. The *t*11 in dairy fat derives from microbial biohydrogenation of PUFA in the rumen. Dairy fat also contains CLA, especially *c*9,*t*11 CLA produced by rumen microbes and primarily by endogenous Δ9-desaturation of *t*11 by the mammary gland [23].

It has been argued that in contrast to other *trans *C18:1, intake of *t*11 is not associated with coronary heart disease or inflammation [24]. In fact, *t*11 may even impart health benefits due to its function as a metabolic precursor of *c*9,*t*11 CLA. This conversion also occurs in humans [22,25,26]. Both, *t*11 and *c*9,*t*11 CLA have been shown to exert anti-inflammatory effects in allergy models [27,28], and *t*11 did not negatively alter inflammatory parameters in humans [29]. High maternal dairy fat intake led to elevated *c*9,*t*11 CLA and *t*11, thus decreasing the *t*9/*t*11-index in breast milk [30]. These ruminant fatty acids in breast milk showed protective effects against the development of atopic manifestation in infants [31].

To date, no corresponding data concerning individual *trans *C18:1 such as *t*9 and *t*11 are available for maternal and the corresponding fetal blood. This study aimed at characterising the distribution of individual *trans *isomers of maternal and fetal blood lipids and their association with LC-PUFA in fetal blood lipids. In addition, the relationship between maternal intake of dairy products and the maternal and fetal blood fatty acid profile was investigated.

## Methods

### Subjects and study design

Blood samples from 55 mothers together with cord blood from their neonates were collected at birth. In this study, only healthy infants born at term after uncomplicated pregnancies were included. Mothers with gestational diabetes or those taking pharmaceuticals were excluded. After birth, mothers were requested to answer two questionnaires: one focussing on obstetric history, lifestyle, and socioeconomic factors and the other was a food frequency questionnaire (FFQ) dealing with foods from a variety of groups, such as dairy products, fish, oils, seeds or nuts. The study protocol was approved by the local ethics committee (Nr. 1345-06/04). Informed written consent was obtained from all mothers.

### Sample collection and preparation for gas chromatography

Maternal blood was drawn at birth from a peripheral vein. Fetal blood was drawn by venipuncture from the placental portion of the umbilical cord immediately after clamping. Blood was collected in EDTA-tubes (S-Monovette 9 ml KE3, Sarstedt). Plasma was separated by centrifugation (1500 × g, 10 min, 4°C) and erythrocytes were washed three times with physiological sodium chloride solution. Plasma and erythrocytes were frozen immediately at -80°C and stored until lipid extraction.

### Lipid extraction and transmethylation

Lipids were extracted from plasma and erythrocytes based on the method of Bligh and Dyer [32]. Lipid extracts were transmethylated by using a combination of 0.5 N methanolic sodium hydroxide (Merck) and 10% (w/w, Supelco) boron trifluoride-methanol (100°C for 5 min each). Subsequently, fatty acid methyl esters (FAME) were purified by thin layer chromatography and dissolved in n-hexane for analysis [33]. A system of two GC/FID methods was used to analyse the full fatty acid spectrum from C4 to C26 including CLA (GC-17 V3 Shimadzu; DB-225MS: 60 m, i.d. 0.25 mm, 0.25 μm film thickness; Agilent Technologies) as well as *cis *and *trans *isomers of C18:1, *trans *C18:2 and C18:3 (GC-2010, Shimadzu; CP-select 200 m × 0.25 mm i.d. with 0.25 μm film thickness; Varian) [21]. In brief, injector and detector temperatures were maintained at 260°C and 270°C, respectively, with hydrogen as carrier gas [21]. In total 88 fatty acids were identified and detectable *trans *C18:1 comprise the following isomers: *t*4, *t*5, *t*6/7/8, *t*9, *t*10, *t*11, *t*12, *t*13/14, *t*15, and *t*16 C18:1. Furthermore, *t*9,*t*12; *c*9,*t*12; *t*9,*c*12 C18:2; *t*3,*c*9,*c*11 and *c*8,*t*10,*t*12 C18:3, were summarised as total *t*FA. Fatty acid concentrations were expressed as the percentage of the total area of all FA peaks (% of total FAME).

### Data evaluation and statistics

Evaluation of food frequency questionnaires (n = 41) was carried out using Prodi 5.5 Nutriscience software (summarized in Table 1). Fourteen questionnaires had to be excluded due to unreliable data.

**Table 1:** Daily intake of energy and dietary fatty acids estimated by food frequency questionnaires and regarding calculated dairy fat intake (n = 41)

	**Total (n = 41)**				**Dairy fat intake** **< 40 g/d (n = 27)**		**Dairy fat intake** **> 40 g/d (n = 14)**		**P^1^**
	
**Reported daily dairy fat intake g/d**	**36.7 ± 16.5**				**27.1 ± 7.88**		**56.3 ± 11.8**		**###**
	
Kcal	2544	± 595			2463	± 561	2699	± 649	
Energy intake, MJ/d	10.6	± 2.49			10.3	± 2.35	11.3	± 2.72	
Carbohydrates, g/d (en%)	244	± 60.9	(40.5	± 5.60)	247	± 57.7	237	± 68.6	
Protein, g/d (en%)	106	± 35.4	(17.4	± 3.16)	101	± 32.3	115	± 40.5	
Fat, g/d (en%)	124	± 33.4	(45.5	± 4.74)	116	± 30.1	140	± 34.9	#
SFA, g/d (en%)	53.9	± 14.1	(19.2	± 2.94)	48.3	± 11.5	64.8	± 12.4	###
MUFA, g/d (en%)	45.8	± 13.5	(16.1	± 2.07)	43.5	± 12.1	50.3	± 15.3	
PUFA, g/d (en%)	16.9	± 6.25	(5.90	± 1.36)	17.1	± 6.22	16.7	± 6.54	
n-3 PUFA, g/d	1.67	± 0.54			1.50	± 0.53	2.00	± 0.38	##
n-6 PUFA, g/d	10.2	± 3.97			9.88	± 3.64	10.8	± 4.64	
Short chain FA, g/d	2.11	± 1.02			1.54	± 0.60	3.20	± 0.72	###
Medium chain FA, g/d	1.88	± 0.74			1.51	± 0.56	2.58	± 0.52	###
Long chain FA, g/d	78.1	± 25.8			68.7	± 20.8	96.2	± 25.5	##
LC-PUFA, g/d	0.42	± 0.26			0.41	± 0.28	0.45	± 0.21	
n-3 LC-PUFA, g/d	0.32	± 0.22			0.30	± 0.24	0.34	± 0.18	
n-6 LC-PUFA, g/d	0.24	± 0.11			0.23	± 0.12	0.25	± 0.09	
C18:1 *t*11^2^	0.37	± 0.16			0.27	± 0.08	0.56	± 0.12	###

Mean ± SD; SFA, saturated fatty acids; MUFA, monounsaturated fatty acids; PUFA, polyunsaturated fatty acids.

^1 ^Means are significantly different between low and high dairy fat intake (t-Test): # P < 0.05; ## P < 0.01; ### P < 0.001.

^2 ^Calculated with 1% *t*11 of dairy fat [21].

Statistical analysis was performed via PASW statistics, version 17 (SPSS Inc.). To evaluate differences and correlations between maternal and fetal fatty acid compositions, paired student's t-test was conducted and Pearson correlation coefficient was calculated. Unpaired t-test was used to determine the difference of means in the subgroups of high and low dairy fat intake. Data were reported as means ± SD. Significance was defined as P ≤ 0.05.

## Results

### Subjects

The mean maternal age at birth was 29.2 years. Infants had normal birth weight and length (Table 2).

**Table 2:** Maternal and infant characteristics (n = 55 mother-child pairs)

Infant sex: male, n (%)	32 (58.2)	
Gestational age (wk)	39.5 ± 1.07	^1^
Birth weight (g)	3450 ± 476	^1^
Birth length (cm)	50.4 ± 2.50	^1^
Delivery method, n (%)		
Spontaneous labour	31 (56.4)	
Elective caesarean	20 (36.4)	
Caesarean after failure to progress in labour	4 (7.30)	
Parity at entry, n (%)		
0	34 (61.8)	
1	11 (20.0)	
≥2	6 (10.1)	
Maternal age (years)	29.2 ± 5.28	^1^
Maternal BMI before pregnancy (kg/m^2^)	22.3 ± 2.6	^1^
Maternal BMI at birth (kg/m^2^)	27.8 ± 2.84	^1^
Maternal atopy, n (%)	16 (29.1)	
Tobacco exposition (maternal and/or paternal smoking) in pregnancy, n (%)	18 (32.7)	

All blood donors.

^1 ^mean ± SD.

### Dietary intake

Mothers reported that they did not essentially change their dietary habits during pregnancy. In general, they consumed western-style diets that were predominantly omnivore. The fat intake (n = 41) averaged 124 g per day which corresponds to 45% of total energy intake (en%), with high inter-individual variations (from 55 to 215 g/d; Table 1). Mean dairy fat intake was 36.7 g/d, ranging from 7.29 g/d (2.2 en%) to 82.1 g/d (39.3 en%). Mothers who reported a high dairy fat intake (> 40 g/d) tended to have higher energy uptake due to the raised dietary fat (increased by 1.2 times). In addition, their dietary intake of saturated and short chain fatty acids was also 1.3 to 2 times higher (Table 1). Overall, fish was reportedly consumed less than once a week and n-3 PUFA supplementation (e.g. by fish oil capsules) was not common (n = 11; 20%). A high dairy fat intake was not associated with fish oil supplementation and increased intake of n-3 PUFA-rich foods (e.g., fish, nuts, and vegetable oils). If fish oil was supplemented, a higher proportion of n-3 LC-PUFA in maternal lipids was not observed (data not shown).

### Fatty acid profile in maternal and fetal blood lipids (n = 55)

#### - *Trans *fatty acids

The mean total *t*FA, as well as *t*9 and *t*11 in plasma and erythrocyte lipids were significantly higher in maternal compared to fetal lipids (Table 3). However, in both fetal lipids fractions, *t*11 levels were half of *t*9 values leading to a higher fetal *t*9/*t*11-index compared to the maternal index in both plasma and erythrocytes (Table 3).

**Table 3:** Fatty acids in maternal and fetal plasma and erythrocytes (n = 55)

	**Plasma**							**Erythrocytes**						
	
	**Mean ± SD^1^** **(% of total FAME)**					**Correlation^2^** **maternal *vs*. fetal**		**Mean ± SD^1^** **(% of total FAME)**					**Correlation^2^** **maternal *vs*. fetal**	
	
	**maternal**		**fetal**		**P**	**r**	**P**	**maternal**		**fetal**		**P**	**r**	**P**
								
C14:0	1.21	± 0.40	1.12	± 0.34		0.26		0.83	± 0.51	0.63	± 0.30	*	-0.06	
C15:0	0.27	± 0.06	0.20	± 0.05		0.31	•	0.25	± 0.07	0.17	± 0.03		0.27	•
C16:0	30.2	± 2.31	29.8	± 1.40		0.15		31.8	± 2.86	34.9	± 1.62	***	0.18	
C17:0	0.24	± 0.04	0.26	± 0.05		0.24		0.35	± 0.12	0.29	± 0.04		0.30	•
C18:0	5.59	± 0.78	9.99	± 0.81	***	0.12		15.1	± 1.51	15.3	± 1.18		0.01	
C18:1 n-9	23.9	± 2.45	18.5	± 2.01	***	0.35	••	17.5	± 1.10	13.8	± 1.01	***	0.16	
C18:1 n-7	2.05	± 0.26	3.50	± 0.46	***	0.45	•••	1.51	± 0.16	2.73	± 0.31	***	0.42	•••
C18:2 n-6	23.5	± 3.41	10.9	± 1.59	***	0.35	••	10.5	± 1.08	4.55	± 0.52	***	0.54	•••
C18:3 n-6	0.16	± 0.06	0.30	± 0.06	***	0.32	•	0.05	± 0.03	0.05	± 0.03		-0.20	
C20:3 n-6	1.55	± 0.32	3.31	± 0.59	***	0.29	•	1.92	± 0.43	3.22	± 0.50	***	0.35	••
C20:4 n-6 AA	3.83	± 0.89	11.7	± 1.94	***	0.29	•	10.6	± 2.47	15.0	± 1.42	***	0.24	
C22:4 n-6	0.02	± 0.02	0.03	± 0.06		-0.05		0.03	± 0.03	0.03	± 0.06		0.07	
C22:5 n-6	0.19	± 0.08	0.53	± 0.16	***	0.66	•••	0.56	± 0.23	1.08	± 0.24	***	0.60	••
C18:3 n-3	0.41	± 0.11	0.12	± 0.04	***	0.37	••	0.15	± 0.04	0.03	± 0.03	***	0.04	
C20:5 n-3 EPA	0.24	± 0.10	0.25	± 0.14		0.15		0.33	± 0.16	0.13	± 0.06	***	0.32	•
C22:5 n-3	0.16	± 0.04	0.21	± 0.09	***	0.17		1.29	± 0.49	0.41	± 0.12	***	-0.02	
C22:6 n-3 DHA	1.21	± 0.35	3.00	± 0.83	***	0.07		3.40	± 1.45	4.24	± 0.98	***	0.02	
SFA	67.8	± 4.77	71.2	± 3.09	***	0.14		80.2	± 6.71	86.3	± 3.24	***	0.05	
MUFA	29.2	± 2.93	26.1	± 2.97	***	0.30	•	19.6	± 1.25	17.2	± 1.11	***	0.16	
n-6 LC-PUFA	5.59	± 1.08	15.6	± 2.14	***	0.25		13.1	± 2.71	19.4	± 1.52	***	0.14	
n-3 LC-PUFA	1.61	± 0.46	3.46	± 0.99	***	0.10		5.02	± 2.04	4.79	± 1.12		0.00	
C18:1 *t*6/7/8	0.03	± 0.01	0.02	± 0.01	***	0.11		0.07	± 0.05	0.07	± 0.09		0.05	
C18:1 *t*9	0.12	± 0.02	0.10	± 0.03	***	0.24		0.15	± 0.03	0.12	± 0.12		-0.06	
C18:1 *t*10	0.05	± 0.02	0.03	± 0.01	***	0.19		0.07	± 0.02	0.05	± 0.08		-0.12	
C18:1 *t*11	0.10	± 0.05	0.05	± 0.03	***	0.51	•••	0.14	± 0.04	0.06	± 0.05	***	0.13	
C18:1 *t*12	0.07	± 0.02	0.03	± 0.01	***	0.29	•	0.11	± 0.03	0.04	± 0.04	***	0.11	
C18:1 *t*13/14	0.05	± 0.02	0.17	± 0.09	***	0.19		0.08	± 0.03	0.20	± 0.04	***	0.23	
C18:1 *t*15	0.03	± 0.01	0.02	± 0.01	**	0.18		0.07	± 0.02	0.03	± 0.03	***	0.16	
C18:1 *t*16	0.05	± 0.02	0.03	± 0.01	***	0.37	••	0.06	± 0.02	0.03	± 0.01	***	0.09	
C18:2 *t*9,12	0.02	± 0.02	0.03	± 0.02	***	-0.24		0.02	± 0.02	0.02	± 0.01		0.09	
Total *t*FA	0.59	± 0.12	0.52	± 0.17	**	0.36	••	0.82	± 0.15	0.64	± 0.45	**	0.07	
*t*9/*t*11-index	1.30	± 0.53	2.17	± 0.86	***	0.39	•••	1.11	± 0.33	2.13	± 0.64	***	0.36	••
*c*9,*t*11 CLA	0.20	± 0.07	0.14	± 0.04	***	0.84	•••	0.12	± 0.04	0.08	± 0.04	***	0.32	•

FAME, fatty acid methyl esters; SFA, saturated fatty acids (sum of C12:0, C14:0, C15:0, C16:0, C17:0 and C18:0); MUFA, monounsaturated fatty acids (sum of the *cis *isomers of C16:1, C18:1 n-9 and C18:1 n-7); n-3 and n-6 LC-PUFA, omega-3 and omega-6 long chain polyunsaturated fatty acids (sum of C20:5 n-3, C22:5 n-3, C22:6 n-3 and C20:3 n-6, C20:4 n-6, C22:4 n-6, C22:5 n-6, respectively); total *t*FA, total *trans *fatty acids (sum of C18:1 *t*4, *t*5, *t*6/7/8, *t*9, *t*10, *t*11, *t*12, *t*13/14, *t*15 and *t*16 as well as C18:2 *t*9,12, C18:3 *t*3,*c*9,*c*11 and C18:3 *c*8,*t*10,*t*12).

^1 ^Means are significantly different between maternal and fetal lipids (paired t-Test): *P < 0.05; ** P < 0.01; *** P < 0.001.

^2 ^r = Pearson correlation coefficient between the respective fatty acid in maternal and fetal plasma or erythrocyte lipids: • P < 0.05; •• P < 0.01; ••• P < 0.001.

Total *t*FA in maternal plasma (P_mat_) correlated positively with total *t*FA concentrations in fetal plasma (P_fet_). There was no correlation of *t*9 between P_mat _and P_fet_. On the contrary, there was a positive correlation between P_mat _and P_fet _for *t*11. In general, no correlation for total *t*FA as well as *t*9 and *t*11 was found between maternal and fetal erythrocytes (E_mat_, E_fet_, respectively; Table 3).

On the other hand, positive correlations were found within the respective blood fraction between *t*9 and *t*11 (P_mat_: r = 0.33, P < 0.05; P_fet_: r = 0.39, P < 0.001; E_mat_: r = 0.12, P = 0.37; E_fet_: r = 0.89, P < 0.001; data not shown).

In general, in most samples, proportions of *t*4 and *t*5 C18:1 as well as of *t*3,*c*9,*c*11 C18:3 were below the detection limit. The co-eluting isomers *t*13 and *t*14 C18:1 were the only *trans *isomers which were lower in maternal than in fetal lipids (Table 3). [However, since there was no pre-separation of *cis *C18:1 via Ag+-TLC before GC analysis, an overestimation of *t*13/14 C18:1 due to co-elution with *cis *C18:1 isomers *c*6-8 could have occurred [34]. It is also possible that *c*6-8 C18:1 are especially relevant in fetal lipids, however, no data are available in the literature].

#### - Conjugated linoleic acids

The *c*9,*t*11 CLA was significantly higher in maternal than in fetal lipids (Table 3). Moreover, positive correlations of *c*9,*t*11 CLA between maternal and fetal lipids were stronger for plasma than for erythrocytes (Table 3).

In P_mat _and P_fet_, elevated *c*9,*t*11 CLA were seen compared to *t*11, whilst both were equally distributed in the respective erythrocyte lipids. In addition, maternal *t*11 in plasma and erythrocytes was positively correlated to the respective fetal *c*9,*t*11 CLA (r = 0.51, r = 0.59; P < 0.001, respectively, data not shown).

#### - Polyunsaturated fatty acids

Quantities of AA, n-6, and DHA, n-3, were significantly higher in P_fet _and E_fet _than in the respective maternal lipids. In contrast, proportions of linoleic acid (C18:2 n-6) were higher in the maternal than in fetal lipids (Table 3).

#### - Correlation between *trans *fatty acids and polyunsaturated fatty acids

Analysis of correlations between *t*FA and LC-PUFA revealed a heterogeneous result. However, a significant negative association was found in P_fet _between *t*9 and n-3 LC-PUFA (total, DPA n-3, DHA) while *t*11 was negatively associated with n-6 LC-PUFA in E_mat _and E_fet _(Table 4).

**Table 4:** Correlation (r) between *t*FA and LC-PUFAs in maternal and fetal plasma and erythrocytes (n = 55)

		n-6				n-3						n-6 LC-PUFA		n-3 LC-PUFA	
						
		C20:4 n-6 AA		C22:5 n-6 DPA		C20:5 n-3 EPA		C22:5 n-3 DPA		C22:6 n-3 DHA					
**A) Between different fatty acids within the same blood fraction (e.g., P_mat _to P_mat_)**															

C18:1 *t*9	P_mat_	-0.33	•	-0.28	•	-0.11		-0.18		-0.28	•	-0.33	•	-0.26	
	P_fet_	-0.21		-0.07		-0.08		-0.40	••	-0.36	••	-0.24		-0.35	••
	E_mat_	0.13		-0.18		-0.05		0.18		0.03		0.13		0.06	
	E_fet_	-0.26		0.03		-0.06		0.02		0.01		-0.26		0.00	

C18:1 *t*11	P_mat_	-0.08		0.05		0.25		-0.19		0.06		-0.12		0.11	
	P_fet_	-0.24		0.02		0.07		0.06		-0.01		-0.22		0.01	
	E_mat_	-0.32	•	-0.29	•	0.07		-0.06		-0.13		-0.36	••	-0.10	
	E_fet_	-0.31	•	-0.01		0.04		0.14		0.10		-0.30	•	0.11	

total *t*FA	P_mat_	-0.25		0.04		0.16		-0.24		-0.07		-0.22		-0.01	
	P_fet_	-0.22		-0.03		0.06		-0.04		-0.09		-0.21		-0.07	
	E_mat_	-0.30	•	-0.31	•	-0.02		-0.05		-0.16		-0.32	•	-0.13	
	E_fet_	-0.30	•	0.01		-0.07		0.00		-0.02		-0.29	•	-0.02	

**B) Between different maternal and fetal fatty acids within the respective blood fraction (e.g., P_mat _to P_fet_)**															

C18:1 *t*9	P_mat _→ P_fet_	-0.14		-0.14		0.00		-0.05		-0.15		-0.17		-0.13	
C18:1 *t*11		-0.19		0.05		0.10		0.21		0.08		-0.20		0.10	
total *t*FA		-0.24		-0.05		0.09		0.08		-0.08		-0.21		-0.05	

C18:1 *t*9	E_mat _→ E_fet_	0.15		-0.11		-0.19		-0.12		-0.04		0.08		-0.06	
C18:1 *t*11		-0.09		-0.16		0.46	••	0.41	••	0.26		-0.12		0.29	•
total *t*FA		-0.01		-0.17		0.29	•	0.24		0.20		-0.03		0.21	

Pearson correlation coefficient (r); • P < 0.05; •• P < 0.01.

**A) **e.g. *t*9 P_mat _to AA P_mat._

**B) **e.g. *t*9 P_mat _to AA P_fet._

Furthermore, regarding correlations of maternal to fetal fatty acids, *t*11 in maternal blood lipids was positively associated with fetal n-3 LC-PUFA, especially in erythrocytes (Table 4(B)).

### Association between dairy fat intake and fatty acid profile of blood lipids (n = 41)

A high maternal intake of dairy fat (> 40 g/d; n = 14) resulted in an elevated amount of milk specific fatty acids such as C15:0, C17:0 and *t*11 in both maternal lipid fractions (P_mat _and E_mat_) compared to mothers with lower dairy fat intake (n = 27; Table 5). In contrast, in fetal lipids, the high maternal dairy fat intake was only reflected by a higher *c*9,*t*11 CLA in plasma and erythrocytes, whereas *t*11 was not elevated. But, since *t*11 was elevated in maternal lipids, the *t*9/*t*11-index had decreased, which was also shown in fetal lipids. In addition, due to a high dairy fat intake, n-3 LC-PUFA such as EPA, DPA, and DHA were elevated in fetal lipids, particularly in plasma (Table 5).

**Table 5:** Differences of selected fatty acids in maternal and fetal plasma and erythrocytes regarding high and low dairy fat intake

			Plasma						Erythrocytes							
		
		Dairy fat intake	maternal^1^			fetal^1^			P^2^	maternal^1^			fetal^1^			P^2^
					
SFA	C15:0	high	0.30	± 0.07	#	0.21	± 0.04	‡	***	0.31	± 0.09	#	0.18	± 0.02		***
		low	0.26	± 0.05		0.19	± 0.04		***	0.24	± 0.05		0.16	± 0.03		***
	C17:0	high	0.26	± 0.05	#	0.26	± 0.03			0.38	± 0.08		0.30	± 0.04		***
		low	0.23	± 0.04		0.25	± 0.05			0.36	± 0.17		0.29	± 0.04		*
n-6	C18:2 n-6	high	22.7	± 2.89	#	10.8	± 1.09		***	9.77	± 0.89		4.40	± 0.45		***
		low	23.8	± 3.58		10.8	± 1.45		***	10.7	± 1.08		4.56	± 0.49		***
	C20:4 n-6 AA	high	4.15	± 0.99		11.6	± 1.63		***	9.90	± 2.81	‡	15.3	± 1.34		***
		low	3.64	± 0.86		11.6	± 2.21		***	10.8	± 2.41		14.9	± 1.62		***
	n-6 LC-PUFA	high	5.90	± 1.22		15.4	± 1.83		***	12.2	± 3.15		19.7	± 1.46		***
		low	5.38	± 1.05		15.5	± 2.49		***	13.3	± 2.63		19.2	± 1.66		***

n-3	C20:5 n-3 EPA	high	0.30	± 0.12		0.29	± 0.10	##		0.34	± 0.16	#	0.17	± 0.06		***
		low	0.21	± 0.08		0.24	± 0.17			0.33	± 0.14		0.12	± 0.04		***
	C22:5 n-3 DPA	high	0.17	± 0.06		0.24	± 0.05	##	**	1.21	± 0.48		0.47	± 0.10	#	***
		low	0.16	± 0.05		0.19	± 0.07		*	1.30	± 0.44		0.38	± 0.08		***
	C22:6 n-3 DHA	high	1.34	± 0.32		3.17	± 0.72	‡	***	3.21	± 1.64	‡	4.61	± 0.84		**
		low	1.16	± 0.38		2.86	± 0.72		***	3.55	± 1.41		4.11	± 0.96		§
	n-3 LC-PUFA	high	1.81	± 0.44		3.70	± 0.84	#	***	4.76	± 2.25	‡	5.25	± 0.97		
		low	1.52	± 0.48		3.29	± 0.83		***	5.17	± 1.92		4.61	± 1.05		

*t*FA	C18:1 *t*9	high	0.12	± 0.02		0.09	± 0.01		***	0.15	± 0.03		0.10	± 0.02		***
		low	0.12	± 0.03		0.10	± 0.03		**	0.15	± 0.03		0.15	± 0.17		
	C18:1 *t*10	high	0.05	± 0.01		0.03	± 0.01		***	0.07	± 0.02		0.04	± 0.02		***
		low	0.05	± 0.02		0.02	± 0.01		***	0.07	± 0.02		0.07	± 0.12		
	C18:1 *t*11	high	0.12	± 0.04	#	0.05	± 0.02		***	0.17	± 0.04	#	0.06	± 0.03		***
		low	0.09	± 0.03		0.05	± 0.02		***	0.13	± 0.02		0.07	± 0.06		***
	C18:1 *t*12	high	0.08	± 0.02	##	0.03	± 0.01		***	0.13	± 0.03		0.04	± 0.01		***
		low	0.07	± 0.02		0.03	± 0.01		***	0.10	± 0.02		0.05	± 0.06		***
	total *t*FA	high	0.62	± 0.09	#	0.49	± 0.10		***	0.92	± 0.19	#	0.57	± 0.13		***
		low	0.56	± 0.10		0.50	± 0.15			0.78	± 0.10		0.74	± 0.64		
	*t*9/*t*11-	high	1.07	± 0.31	#	1.81	± 0.35		***	0.91	± 0.28	#	1.74	± 0.58	#	***
	index	low	1.42	± 0.48		2.32	± 0.83		***	1.18	± 0.29		2.35	± 0.70		***

CLA	*c*9,*t*11 CLA	high	0.25	± 0.06	##	0.17	± 0.04	##	***	0.15	± 0.05	#	0.11	± 0.06	‡	
		low	0.18	± 0.04		0.12	± 0.03		***	0.10	± 0.03		0.07	± 0.02		***

^1 ^Portions of the respective fatty acid in % of total FAME as mean ± SD of high dairy fat intake (> 40 g/d, upper values; n = 14) and low dairy fat intake (< 40 g/d, lower values; n = 27); means are different between both groups (unpaired t-Test): ‡ P < 0.1; # P < 0.05; ## P < 0.01.

^2 ^Means are different between maternal and fetal lipids in the respective group of dairy fat intake (paired t-Test): § P < 0.1 * P < 0.05; ** P < 0.01; *** P < 0.001.

**Figure 1:**
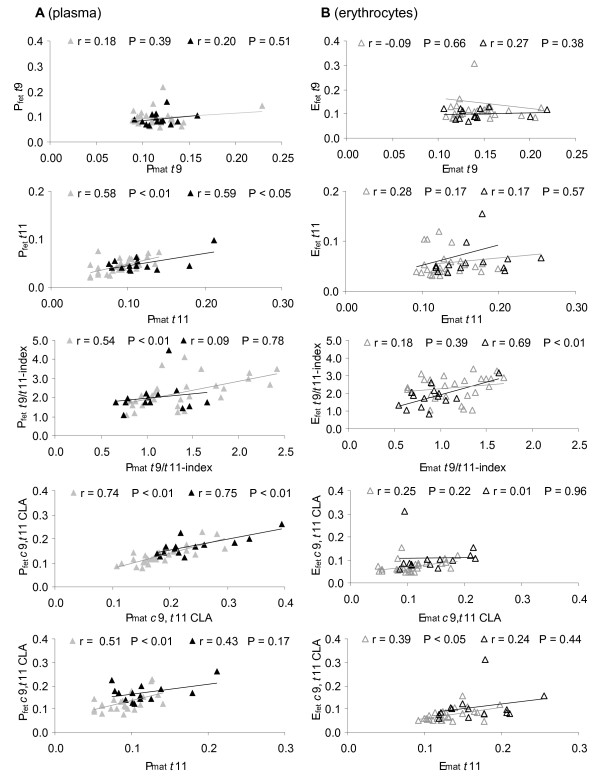
**Correlation of the individual *t*FA, the *t*9/*t*11-index, and *c*9,*t*11 CLA**. Correlation between the respective fatty acid in maternal and fetal (A) plasma (filled signs) and (B) erythrocytes (open signs) in groups of low (< 40 g/d, n = 27; grey) and high (> 40 g/d, n = 14; black) dairy fat intake. Correlations irrespective of dairy fat intake are listed in Table 3.

Data analysis regarding fatty acid correlation between maternal and fetal lipids clearly showed that both *t*11 and *c*9,*t*11 CLA were positively correlated between P_mat _and P_fet _in the case of low and high dairy fat intake (Figure 1). In contrast, no correlation was observed for *t*9 between maternal and fetal lipids, independently of dairy fat intake (Figure 1). A high dairy fat intake did not result in a negative correlation between *t*11 and *t*9 in all maternal and fetal blood lipids (data not shown).

## Discussion

The quantities of fatty acids in maternal and, especially, in fetal blood are under the influence of various factors. Since *t*FA are not synthesised in human tissue, all *t*FA with isolated double bonds present in fetal blood lipids can only be due to a transplacental transfer and must originate from maternal diet. Herein, we investigated the fatty acid composition of plasma and erythrocytes in mother-child pairs at birth, focussing on total *t*FA and individual *trans *C18:1 such as *t*9 and *t*11 with special regard to dairy fat intake.

### -Total *trans *fatty acids in maternal and fetal blood lipids

In the present study, total *t*FA concentrations in maternal and fetal lipids were about 0.5 to 0.8% of FAME, with generally higher levels in maternal and plasma lipids compared to fetal and erythrocyte lipids, respectively. In several recently published European studies, values for total *t*FA in maternal and fetal erythrocytes and plasma lipids range from 0.08% to 0.45% in phospholipids and up to 2.74% in the other lipid fractions, however, with higher *t*FA values in maternal than in fetal lipids [13,19,20,35]. Nevertheless, comparison of data for total *t*FA is difficult since percentages of single fatty acids may vary according to the analysed plasma fractions (phospholipids *vs*. total lipids; [36]) and the applied method of analysis (GC-column, varying numbers of analysed/identified fatty acids, integration parameters, management of un-identified peaks).

### - *trans *fatty acid intake

Following a number of accounts regarding adverse health effects [6,37], the *t*FA, content in foods has continuously decreased over the last decades [21,38]. Recent reports estimate the mean dietary *t*FA intake in Germany at 2.3 g/d for men (0.8 en%) and 1.6 g/d for women (0.74 en%; [39]). Since *t*FA content varies strongly within food categories [21], the exact intake of *t*FA is difficult to calculate. Furthermore, the distribution of individual *t*FA isomers varies according to their origin. In dairy fat, *t*11 is generally the major *t*18:1 containing about 1.0 to 2.0% of FAME [21,40]. Thus, the present maternal mean values for low and high dairy fat intake were approximately 27.1 and 56.3 g/d, which were estimated as corresponding to about 0.3 to 0.6 g *t*11/d, respectively (Table 2). This assessment is in line with recent data regarding the average *t*11 intake [39]. In contrast, the *t*9 intake in the age group 30 ± 5 years of the present study population was found to be about twice as high as the *t*11 intake [39] resulting in a mean dietary *t*9/*t*11-index of about 2.

### -Individual *trans *fatty acids in blood lipids

Hardly any data are available in the literature concerning individual *trans *C18:1 such as *t*11 and *t*9 in maternal and/or fetal blood lipids. The present proportions of *t*9 and *t*11 were similar in maternal blood lipids (P_mat _0.12 *vs*. 0.10). Whereas *t*9 in fetal blood lipids tended to be lower compared to maternal lipids, *t*11 was only half the value of maternal *t*11 (P_mat _0.10 *vs*. P_fet _0.05; Table 3). This resulted in fetal *t*9/*t*11-indices being twice as high as maternal indices (Table 3, 5).

A general positive correlation of *t*11, but not of *t*9 was seen between maternal and fetal plasma (Table 3 Figure 1). However, fetal *t*11 was about the half that of *t*9 (Table 3). This low value could indicate differences in materno-fetal transfer as well as metabolism (oxidation, conversion, incorporation) of both individual *trans *isomers *t*9 and *t*11, as has been observed in rat hepatocytes [41,42].

### -Conjugated linoleic acids in the diet and blood lipids

Dairy fat contains CLA (mainly *c*9,*t*11 CLA), which is formed by Δ9-desaturation of *t*11 [23]. In dairy fat and human breast milk, *t*11 was generally higher compared to *c*9,*t*11 CLA, whilst the ratio ranged from 4:1 to 2:1 [31,43,44].

In the present study, irrespective of dairy fat intake, *t*11 was lower compared to *c*9,*t*11 CLA in lipids of maternal and fetal plasma (1:2; Table 3) probably reflecting the conversion of *t*11 to *c*9,*t*11 CLA by Δ9-desaturase and/or its preferred oxidation [22]. In contrast, the ratio of *t*11 to *c*9,*t*11 CLA in erythrocyte lipids was about 1:1, since maternal and fetal *c*9,*t*11 CLA was lower compared to their levels in plasma, in accordance with data described by Mueller et al [17]. This aspect may be caused by a higher incorporation of CLA into neutral lipids than into phospholipids, the major lipid fraction in erythrocytes [45]. Further, our results confirmed that maternal *c*9,*t*11 CLA (dietary and of endogenous origin) was positively correlated to fetal *c*9,*t*11 CLA (Table 3) [44].

### -Impact of high dairy fat intake on composition of fatty acids in blood lipids

The present results clearly showed an association between ruminant fat consumption and fatty acid distribution of human lipids. A high dairy fat intake in mothers (> 40 g/d) resulted in increased *c*9,*t*11 CLA and total *t*FA, especially *t*11 in blood lipids. Moreover, the raised *t*11 concentrations resulted in a decreased maternal *t*9/*t*11-index (Table 5).

In breast milk of mothers with comparably high consumption of dairy fat (> 40 g/d), elevated *t*11 and *c*9,*t*11 CLA and a decreased *t*9/*t*11-index were also observed [30,44]. The increase in the milk specific fatty acids C15:0 and C17:0 in maternal blood also confirmed the high dairy fat intake in the present study (Table 5). Similar results were obtained in breast milk in a former study [44]. Interestingly, due to a maternal high dairy fat intake, only *c*9,*t*11 CLA and not *t*11 was elevated in the corresponding fetal blood lipids (Table 5).

However, there was a positive correlation of *t*11 in plasma between mother and child (not in erythrocytes, Figure 1). The *t*9 levels did not significantly differ and there was no correlation between maternal and fetal lipids on comparing a high and low dairy fat diet (Table 5). Although the results point to differences between *t*9 and *t*11 regarding metabolism and/or transfer, respective data are not yet available for humans.

### - *Trans *fatty acids and long chain-PUFA in maternal and fetal blood lipids

The composition of maternal dietary fat and body fat depot [14,35] strongly accounts for the fatty acid composition in fetal tissue. However, the increased amounts of n-6 LC-PUFA (AA, DPA) and n-3 LC-PUFA (DPA, DHA) in fetal plasma and erythrocytes compared to maternal lipids observed in the present and former studies is physiologically consistent and attributed to the special requirements of the fetus (Table 3) [46,47]. Further, a wide range of fatty acid transporters and binding proteins in the placenta determines the enrichment of these LC-PUFA [11,12].

Several publications show an inverse association between total *t*FA and both n-6 (e.g. AA) as well as n-3 LC-PUFA (e.g. DHA) in blood lipids. However, since these data only deal with either maternal or fetal blood lipids and not with their interaction as well as different lipid fractions were used, comparison is difficult [18,20]. In the present study, total *t*FA were also inversely associated with the sum of n-6, but not n-3 LC-PUFA within the same blood fraction (partly significant; Table 4(A)). Regarding n-3 LC-PUFA in fetal plasma, a negative correlation was only found for *t*9, but not for *t*11 (Table 4(A). This result might be of relevance, since n-3 have been shown to extend anti-inflammatory effects [5].

Concerning the relationship between maternal and fetal lipids, the current study data showed a positive correlation of *t*11 to n-3 LC-PUFA, however, the result was only significant in erythrocytes (Table 4(B)). This effect could be associated with a higher dairy fat intake, since n-3 LC-PUFA (EPA, DPA, DHA) were significantly elevated in the respective fetal plasma (Table 5). Some studies have revealed the ability of *t*FA to inhibit several enzymes involved in LC-PUFA synthesis but without distinguishing between industrial and ruminant *t*FA [48,49]. However, there is little data regarding the impact of *t*FA on placental fatty acid transporters showing that *t*9 may inhibit binding of PUFA at placental membranes [50].

Differences between *t*9 and *t*11 indicated herein might be the result of isomer-specific influences on transcription factors (such as PPARs) involved in the expression of placental transport proteins [51,52]. From cellular and animal models, there is evidence that *t*9 inhibits [53] and *t*11 activates [54] PPAR expression. Thus, differences in PPAR activation by *t*9 and *t*11 could interfere with the cellular uptake of LC-PUFA into the placenta [55,56].

## Conclusions

We analysed for the first time individual isomers of *trans *C18:1 in blood lipids of mother-child pairs at birth and found differences between *t*9 and *t*11.

In fetal lipids, *t*11 accumulation was only half that of *t*9, probably due to *t*11 conversion to *c*9,*t*11 CLA. In general, total *t*FA, including *t*9 and *t*11, were higher in maternal than in fetal lipids. The present results also demonstrated that maternal high dairy fat intake led to increased *t*11 and *c*9,*t*11 CLA in the blood lipids. However, *t*11 was not increased in the respective fetal lipids. In addition, the essential n-3 LC-PUFA were elevated in fetal blood lipids.

Thus, *t*FA have to be distinguished according to their origin (ruminant or industrial) to separately investigate their possible influence on fetal development and human health.

## Abbreviations

AA: arachidonic acid; CLA: conjugated linoleic acids; DHA: docosahexaenoic acid; DPA: docosapentaenoic acid; en%: energy %; E_mat _and E_fet_: maternal and fetal erythrocytes; EPA: eicosapentaenoic acid; FAME: fatty acid methyl esters; MUFA: monounsaturated fatty acids; n-3 and n-6 LC-PUFA: omega-3 and omega-6 long chain polyunsaturated fatty acids; P_mat _and P_fet_: maternal and fetal plasma; SFA: saturated fatty acids; *t*FA: *trans *fatty acids; *t*9: *trans*-9 C18:1 (elaidic acid); *t*11: *trans*-11 C18:1 (vaccenic acid).

## Competing interests

The authors declare that they have no competing interests.

## Authors' contributions

UE, LS, GJ, and ES designed the study, UE and LS collected the samples. AJ and KK performed fatty acid analysis. UE performed the Prodi evaluation and the statistical analyses. UE, KK and AJ wrote the paper. All authors read and approved the final manuscript.
